# Vacuum‐Deposited Inorganic Perovskite Light‐Emitting Diodes with External Quantum Efficiency Exceeding 10% via Composition and Crystallinity Manipulation of Emission Layer under High Vacuum

**DOI:** 10.1002/advs.202206076

**Published:** 2023-02-07

**Authors:** Chung‐An Hsieh, Guang‐Hsun Tan, Yung‐Tang Chuang, Hao‐Cheng Lin, Po‐Ting Lai, Pei‐En Jan, Bo‐Han Chen, Chih‐Hsuan Lu, Shang‐Da Yang, Kai‐Yuan Hsiao, Ming‐Yen Lu, Li‐Yin Chen, Hao‐Wu Lin

**Affiliations:** ^1^ Department of Photonics National Yang MingChiao Tung University No. 1001 University Road Hsinchu 300 Taiwan; ^2^ Department of Materials Science and Engineering National Tsing Hua University Hsinchu 30013 Taiwan; ^3^ Institute of Photonics Technologies National Tsing Hua University Hsinchu 30013 Taiwan

**Keywords:** inorganic halide perovskite, light‐emitting diodes, passivation, small molecule, vacuum deposition

## Abstract

Although vacuum‐deposited metal halide perovskite light‐emitting diodes (PeLEDs) have great promise for use in large‐area high‐color‐gamut displays, the efficiency of vacuum‐sublimed PeLEDs currently lags that of solution‐processed counterparts. In this study, highly efficient vacuum‐deposited PeLEDs are prepared through a process of optimizing the stoichiometric ratio of the sublimed precursors under high vacuum and incorporating ultrathin under‐ and upper‐layers for the perovskite emission layer (EML). In contrast to the situation in most vacuum‐deposited organic light‐emitting devices, the properties of these perovskite EMLs are highly influenced by the presence and nature of the upper‐ and presublimed materials, thereby allowing us to enhance the performance of the resulting devices. By eliminating Pb° formation and passivating defects in the perovskite EMLs, the PeLEDs achieve an outstanding external quantum efficiency (EQE) of 10.9% when applying a very smooth and flat geometry; it reaches an extraordinarily high value of 21.1% when integrating a light out‐coupling structure, breaking through the 10% EQE milestone of vacuum‐deposited PeLEDs.

## Graphical abstract

By cleverly introducing vacuum‐sublimed ultrathin upper‐ and under‐layer adjacent to inorganic perovskite emission layer to inhibit Pb^0^ trap forming and enhance radiative recombination, all‐vacuum deposited perovskite light emission diodes are demonstrated with record‐high external quantum efficiencies up to 10.9% and 21.1% without and with a light out‐coupling structure, respectively.

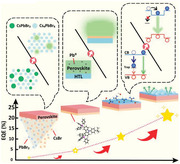

## 1 Introduction

Metal halide perovskites continue to be the subjects of tremendous research and development efforts because of their excellent optoelectronic properties, including high absorption coefficients, tunable bandgaps, and high carrier mobilities.^[^ ^1^, ^2^, ^3^, ^4^, ^5^ ^]^ They have been used with great success in such devices as photovoltaics, photodetectors, light‐emitting diodes (LEDs), and lasers.^[^ ^6^, ^7^, ^8^, ^9^, ^10^, ^11^, ^12^, ^13^, ^14^, ^15^, ^16^, ^17^ ^]^ Because LEDs based on perovskite quantum dots and thin films possess very narrow emission spectral widths, high emission luminance, and tunable emission spectral ranges, they are promising candidates for application in next‐generation high‐color‐gamut display technology.^[^ ^18^, ^19^, ^20^ ^]^ Since the first perovskite LED (PeLED) was reported in 2014, their performance has increased rapidly, recently achieving external quantum efficiencies (EQEs) of up to 20%.^[^ ^21^, ^22^, ^23^, ^24^, ^25^, ^26^ ^]^ At present, high‐performance PeLEDs are fabricated mainly through solution processing. With developments in solvent engineering and composition tuning, the luminescence efficiencies and stabilities of these devices have improved greatly.^[^ ^13^, ^23^, ^24^, ^25^ ^]^ Nevertheless, the quality of a solution‐processed perovskite film is highly sensitive to its fabrication conditions, including the atmosphere, temperature, and annealing process; together with a lack of large‐area manufacturing methods, the mass production of reproducible solution‐processed PeLEDs has been hindered.^[^ ^27^, ^28^ ^]^ With the great success of organic LEDs (OLEDs), which are manufactured predominantly through vacuum deposition, rather than solution methods, it is expected that highly efficient all‐vacuum‐deposited PeLEDs would also inherit the advantages of vacuum processing: layer‐by‐layer stacking giving more capable structures, fabrication of meter‐sized substrates, precise control over the layer thickness and uniformity, and batch‐to‐batch reproducibility.^[^ ^29^, ^30^, ^31^ ^]^ Nevertheless, intriguingly, even with great efforts exerted by many researchers, the efficiency and brightness of vacuum‐processed PeLEDs have lagged those of their solution‐made counterparts. The highest EQEs of vacuum‐deposited PeLEDs have typically been 5–8%, but they have usually been associated with low maximum brightness.^[^ ^32^, ^33^, ^34^, ^35^, ^36^, ^37^, ^38^ ^]^ We believe that the relatively low device performance of vacuum‐deposited PeLEDs has arisen from the complicated nature of the perovskite emission layer (EML) formed under high vacuum. In OLEDs, the EMLs are usually amorphous thin films of composites of vacuum codeposited small molecular hosts and emission dopants. No chemical reactions occur between these two sublimed species. Although the EMLs of vacuum‐deposited PeLEDs feature organic [e.g., methylammonium bromide (MABr)] or inorganic [e.g., cesium bromide (CsBr)] halides codeposited with lead halides [e.g., lead bromide (PbBr_2_)], these sublimed materials undergo relatively complicated chemical bonding and form polycrystalline, rather than amorphous, perovskite under high vacuum.

In this study, we investigated the formation dynamics of vacuum‐sublimated perovskite EMLs. We observed the spontaneous development of mixed phases of 3D CsPbBr_3_ and 0D Cs_4_PbBr_6_, which directly affected the emission properties of the perovskite EMLs and, hence, the subsequent device performance. Surprisingly, the compositions and crystal structures of the EMLs were highly influenced by the nature of both the postdeposited upper‐layer and the presublimed under‐layer, in contrast to the situation in OLED fabrication. By introducing suitable ultrathin under‐ and upper‐layers before and after depositing the EML, we could inhibit trap formation and enhance radiative recombination. Through such hierarchical manipulations, we obtained devices achieving an excellent EQE of 10.9% and a current efficiency (CE) of 38.7 cd A^−1^, in the absence of a light out‐coupling structure—the first instance of a vacuum‐deposited PeLED passing through the 10% EQE milestone. Through selection of a suitable sub‐nanometer interfacial layer, we achieved a maximum luminance (*L*_max_) of 75 300 cd m^−2^. Integration of a light out‐coupling hemisphere structure led to a very high EQE of 21.1%, with a CE of 74.7 cd A^−1^. The novel control methods and unprecedented device efficiency reported herein represent breakthroughs in the development of vacuum‐deposited PeLEDs.

## 2 Results and Discussion

**Figure 1:**
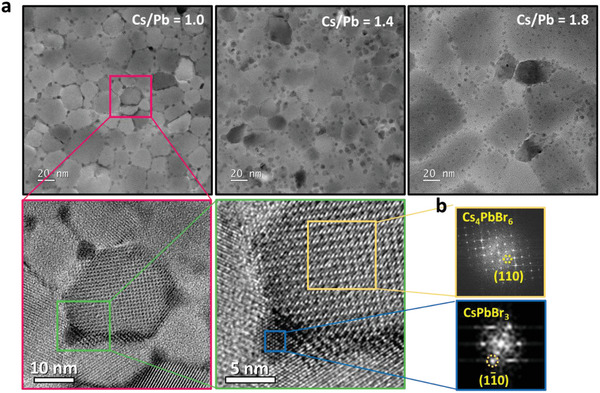
a) TEM images of perovskite thin films fabricated with various CsBr/PbBr_2_ molar ratios. b) FFT patterns of Cs_4_PbBr_6_ and CsPbBr_3_ from selected‐area.

In a previous study of all‐vacuum‐deposited CsPbI_3_ solar cells, we found that it was critical to realize a stoichiometric balance between cesium iodide (CsI) and lead iodide (PbI_2_) to achieve the highest photovoltaic performance.^[^ ^7^ ^]^ Intriguingly, however, vacuum‐deposited perovskite with a carefully balanced stoichiometric ratio exhibited no emission. In contrast, slightly increasing the ratio of CsBr led to a great enhancement in the thin‐film luminance (Figure S1a, Supporting Information), similar to the observation in the previous reports.^[^ ^32^, ^37^, ^39^ ^]^ Figure S1b (Supporting Information) presents the corresponding absorption spectra of 50‐nm‐thick thin films with various CsBr/PbBr_2_ deposition molar ratios. The accurate molar ratios in the thin films were confirmed by energy dispersive spectrometry (EDS) and were very similar to the experimental deposition molar ratios as shown in Figure S2 and summarized in Table S1 in the Supporting Information. All of these films exhibited an absorption onset near 530 nm, the characteristic absorption edge of CsPbBr_3_, indicating its successful formation through coevaporation of CsBr and PbBr_2_ onto the substrate held at room temperature. A very low absorption in the range 400–530 nm appeared for the CsBr/PbBr_2_ = 2.0 sample, suggesting the retarded formation of CsPbBr_3_ at this sublimation ratio.^[^ ^38^ ^]^ An absorption peak near 314 nm appeared in all of the spectra, corresponding to the presence of 0D Cs_4_PbBr_6_. The intensity of this shorter‐wavelength absorption peak increased upon increasing the CsBr molar ratio, implying that a Cs‐rich environment promoted the generation of Cs_4_PbBr_6_. Thus, we suspect that a host/guest system naturally formed, comprising the larger‐band‐gap Cs_4_PbBr_6_ as host and the smaller‐bandgap, but highly emissive, CsPbBr_3_ as guest, analogous to the situation in common OLED EML systems. **Figure** **1**a presents the transmission electron microscopy (TEM) images of the perovskite thin films fabricated with various molar ratios of CsBr/PbBr_2_. The measured *d*‐spacing of 0.42 nm can be indexed as the $\left(1\bar{1}0\right)$ plane of CsPbBr_3_ through fast Fourier transform (FFT) patterns (Figure 1b, CsPbBr_3_) (PDF 01‐086‐3013), while the measured *d*‐spacing of 0.68 nm can be indexed as the (110) plane of Cs_4_PbBr_6_ (Figure 1b, Cs_4_PbBr_6_) (PDF 04‐015‐9683). Therefore, we confirm that the crystals with a larger grain size and a brighter color are Cs_4_PbBr_6_, and the crystals with a smaller grain size and a darker color are CsPbBr_3_. In the meantime, the change of average crystal size of CsPbBr_3_ with the various CsBr/PbBr_2_ molar ratios was observed as shown in Figure 1a. The average crystal domain of CsPbBr_3_ decreased from 4.49 to 2.76 nm upon increasing of the CsBr molar ratio, while the average crystal domain of the Cs_4_PbBr_6_ increased from 25.6 to 38.74 nm, accordingly. This result indicates that Cs_4_PbBr_6_ matrix restricted the size of the CsPbBr_3_ crystals, which also induced a blue‐shift in photoluminescence (PL) occurred in the thin films at higher CsBr ratios due to quantum confinement effect (Figure S1c, Supporting Information). In previous reports of solution‐processed PeLEDs, it has been suggested that Cs_4_PbBr_6_ passivates the surface of CsPbBr_3_ to improve the radiative recombination performance, due to the lattice‐match between CsPbBr_3_ and Cs_4_PbBr_6_.^[^ ^40^ ^]^ We have found, however, that the optimized CsBr/PbBr_2_ ratio was 1.4 (Figure S1a, Supporting Information); together with the lower CsPbBr_3_ absorption in the absorption spectra, we infer that a balance must be struck between the enhanced confinement of the CsPbBr_3_ emitters and their content.

**Figure 2:**
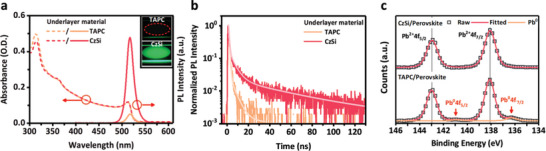
a) Absorption and PL spectra; insets: photographs of UV light‐excited surfaces. b) TRPL and c) Pb 4f XPS spectra of perovskite thin films prepared with different under‐layers.

Having determined the optimized stoichiometric ratio of the vacuum co‐deposited EML, we investigated the effects of under‐layers on these EMLs. Initially, we assumed that, similar to the behavior of vacuum‐deposited amorphous thin films in OLEDs, the emission properties of the EML would not be sensitive to the presence of different under‐layer materials. To our surprise, however, we found that they dramatically affected the emission intensities of the perovskite EMLs. **Figure** **2**a presents the absorption and PL spectra of 30‐nm‐thick perovskite EMLs deposited on 5‐nm‐thick 4,4′‐cyclohexylidenebis[*N*,*N*‐bis(4‐methylphenyl)benzenamine] (TAPC) and 9‐(4‐*tert*‐butylphenyl)‐3,6‐bis(triphenylsilyl)‐9*H*‐carbazole (CzSi). They all provided similar absorption curves, except for a slightly higher absorbance in the 300–320 nm range for the TAPC sample, attributable to the absorption of TAPC (Figure S3, Supporting Information). In contrast, their PL intensities were very different. Because TAPC is commonly used as a hole transporting layer (HTL) in both OLEDs and PeLEDs, it was our first choice for the HTL of our present PeLEDs.^[^ ^33^, ^41^ ^]^ We found, however, that even at the same EML thickness and with the same material absorbance—meaning that the amount of CsPbBr_3_ was identical—the PL intensities of the CzSi sample was 10 times higher than that of the TAPC sample. Figure 2b displays the corresponding time‐resolved PL (TRPL) spectra. The PL lifetimes of the CzSi sample (24.4 ns) was much longer than that (1.5 ns) of the TAPC sample (Table S2, Supporting Information). We further investigated the carrier density‐dependent recombination dynamics of TAPC/perovskite and CzSi/perovskite by transient absorption (TA) with various excitation fluences. As shown in Table S3 (Supporting Information), we extracted the *b* (bimolecular recombination constant) and *c* (trimolecular recombination constant) by fitting TA spectra under different excitation fluences (Figure S4a–d, Supporting Information) with Equation (1) as follows

(1) $$-\frac{dn\left(t\right)}{dt}=a\cdot n\left(t\right)+b\cdot n{\left(t\right)}^{2}+c\cdot n{\left(t\right)}^{3}$$

which can quantitatively describe the carrier density‐dependent recombination kinetics, and the *a*, representing to the monomolecular recombination constant, that can be extracted from the time‐resolved photoluminescence (TRPL) measurements.^[^ ^42^ ^]^ The fitting results show that the bimolecular recombination constant *b*, representing radiative recombination, of CzSi/perovskite (1.69 × 10^−9^cm^3^ s^−1^) is about 10 times higher than that of the TAPC/perovskite sample (1.54 × 10^−10^cm^3^ s^−1^). The results indicate that the ultrathin under‐layer CzSi strategy is promising to improve the performance of PeLEDs. This evidence with the enhanced PL intensities and longer PL lifetimes for the CzSi sample indicated that the under‐layer inhibited the nonradiative quenching of the EMLs. We used X‐ray photoelectron spectroscopy (XPS) to investigate the cause of the unexpectedly higher PL of the perovskite EML deposited on CzSi, relative to that on TAPC. Figure 2c reveals two peaks for Pb^0^ at 140.9 (Pb^0^ 4f_5/2_) and 136.4 (Pb^0^ 4f_7/2_) eV besides those for Pb^2+^ 4f_5/2_ and Pb^2+^ 4f_7/2_ of the perovskite on the TAPC sample, confirming the presence of metallic Pb^0^, a well‐known deep defect state in perovskites.^[^ ^43^, ^44^, ^45^ ^]^ TAPC tends to degrade to opened cyclohexyl ring fragments upon thermal evaporation, providing lone pairs of electrons for redox reaction with Pb^2+^ species, resulting in the formation of Pb^0^ defects.^[^ ^46^, ^47^ ^]^ In contrast, the XPS spectrum of the perovskite EML formed on the thermally stable CzSi featured no signals for metallic Pb^0^, consistent with its higher PL intensity and longer PL lifetime.^[^ ^48^, ^49^ ^]^

**Figure 3:**
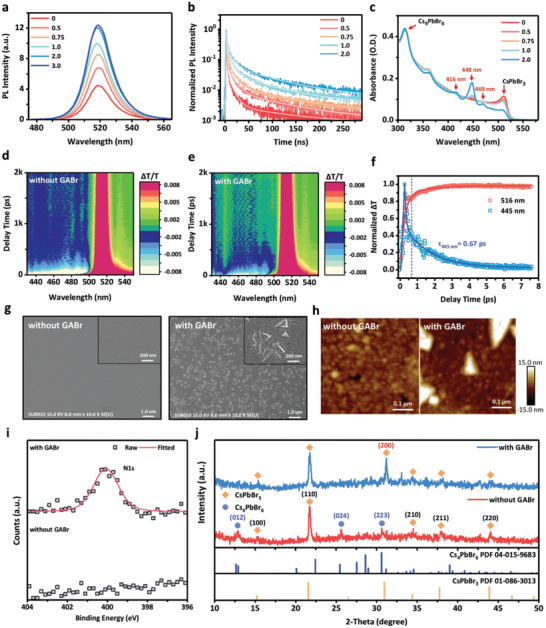
a) PL intensity spectra, b) TRPL spectra, and c) absorption spectra of perovskite films treated with GABr at various concentrations (mg mL^−1^). d,e) Time‐wavelength‐dependent TA color maps of perovskite films prepared with (1 mg mL^‐1^) and without GABr‐treatment. f) Normalized kinetics of the TA signal at 445 and 516 nm for perovskite films prepared with (1 mg mL^‐1^) GABr‐treatment. g) SEM images, h) AFM images, i) N 1s XPS spectra, and j, XRD patterns of perovskite films prepared with (1 mg mL^−1^) and without GABr‐treatment.

In solution‐processed PeLEDs, larger organic halides are commonly introduced to create some 2D structures for EML surface passivation.^[^ ^50^, ^51^, ^52^, ^53^, ^54^ ^]^ Here, after having selected the CzSi under‐layer and optimized the codeposition ratio, we postdeposited a guanidinium bromide (GABr) upper‐layer onto the perovskite EML. Guanidinium (GA^+^) cations readily form hydrogen–halogen bonds with halide ions, thereby effectively passivating surface halide vacancies.^[^ ^55^ ^]^ **Figure** **3**a presents the PL spectra of CzSi/perovskite films postdeposited with different concentrations of GABr. The emission peak of the CzSi/perovskite film was not affected by GABr treatment, indicating that the emission species remained as 3D perovskite. The PL intensity was, however, enhanced by up to three times under the optimal GABr conditions, with a similar trend in the prolonging of the PL lifetime observed in TRPL measurements (Figure 3b). Table S4 (Supporting Information) lists the TRPL time constants of the CzSi/perovskite films treated with the various concentrations of GABr. The PL lifetime (51.9 ns) of the CzSi/perovskite sample treated with GABr at a concentration of 2.0 mg mL^−1^ was much longer than that of the sample prepared without GABr treatment (24 ns). These results represent that GABr is able to efficiently passivate the perovskite films and prevent the undesired quench in the perovskite.

The TRPL data revealed that GABr treatment decreased the nonradiative recombination loss and manifested radiative recombination of the perovskites. Figure 3c presents the absorption spectra of the CzSi/perovskite films that had been treated with GABr. New peaks appeared at 416, 448, and 469 nm, associated with the perovskite quasi‐2D phase, in the samples treated with high concentrations of GABr. 2D/3D hybrid structures, or quasi‐2D structures, are believed to benefit the luminance efficiency of perovskites, due to their wider bandgap providing additional energy transfer routes to the 3D phase.^[^ ^13^, ^53^, ^56^, ^57^ ^]^ The analysis of TA measurements also detailly revealed the dynamics of photoinduced carriers between quasi‐2D and 3D perovskite. As shown in Figure 3d,e, the CzSi/perovskite thin film with GABr treatment showed a bleach peak around 445 nm, which corresponded to the steady‐state absorption features of CzSi/perovskite/GABr thin film (Figure 3c). The results confirm that the formation of quasi‐2D perovskite by introducing GABr into the 3D perovskite. The normalized kinetics of the TA signal at 445 and 516 nm (Figure 3f) elucidated the characteristics of energy transfer from quasi‐2D perovskite to 3D perovskite. The 445 nm TA kinetics of the large bandgap domain showed a faster lifetime (0.67 ps), while the 518 nm TA kinetics of the smaller bandgap domain exhibited a slower decay. The 516 nm TA kinetics reached to its peak value in a delayed time corresponding to the falling time of 445 nm TA kinetics, which indicates the energy transfer from larger bandgap quasi‐2D perovskite to smaller bandgap 3D perovskite, and finally contributes to a radiative recombination.^[^ ^51^, ^53^ ^]^ Scanning electron microscopy (SEM, Figure 3g) and atomic force microscopy (AFM, Figure 3h) revealed the surface morphologies of the CzSi/perovskite films prepared with and without GABr. In contrast to the smooth surface (*R*_q_ = 2.15 nm) of the pristine perovskite, the perovskite treated with GABr exhibited some additional flaky grains (*R*_q_ = 6.66 nm). The XPS spectra (Figure 3i) revealed a strong N 1s peak at 400.1 eV arising from the NH_2_^+^ groups of the GA^+^ cations, confirming the existence of organic GA^+^ on the perovskite thin film. The resulting Br^‐^‐rich surface on the 2D/3D perovskites promoted the passivation of surface defects. Furthermore, an additional peak at of 31.2°, attributable to the (200) plane of CsPbBr_3_, appeared in the X‐ray diffraction (XRD) patterns of the perovskite EMLs that had been treated with GABr (Figure 3j), suggesting that the new flaky plate structures on the thin film surfaces might have comprised additional quasi‐2D structure. The disappearing of XRD signals at 12.6° and 25.4°, respectively attributable to the (012) plane and (024) plane of Cs_4_PbBr_6_ after GABr treatment, was due to the solvent we used in the GABr treatment, 2‐propanol (IPA), which was evidenced by XRD measurement as shown in Figure S5 in the Supporting Information.

**Figure 4:**
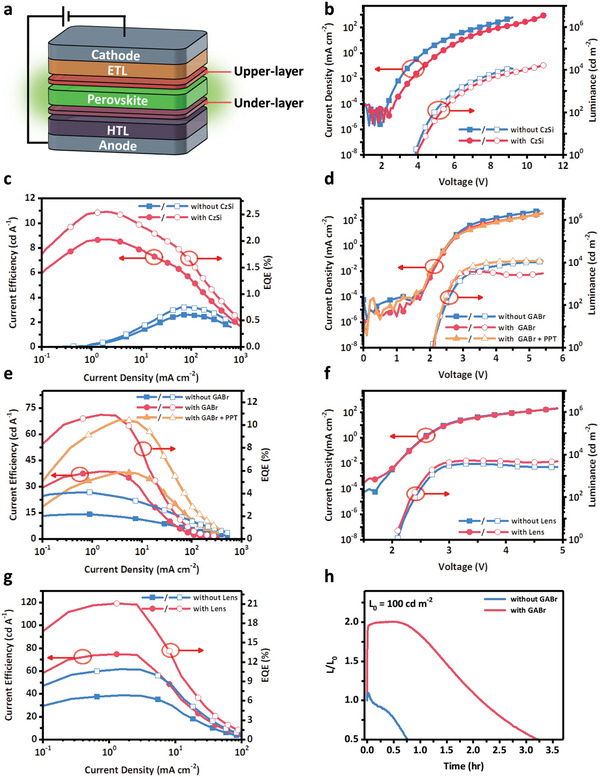
a) Device structure of PeLEDs examined in this study. b) *J*–*V*–*L* characteristics and c) CE and EQE data of PeLEDs prepared with and without CzSi. d) *J*–*V*–*L* characteristics and e) CE and EQE data of PeLEDs prepared without any over‐layers, with GABr, and with GABr and PPT. f) *J*–*V*–*L* characteristics and g) CE and EQE data of PeLEDs prepared with and without a hemisphere light out‐coupling structure. h) Operational lifetimes of PeLEDs prepared with and without GABr, measured at an initial luminance of 100 cd m^−2^.

Encouraged by the these hierarchical enhancements in the PL intensities of the vacuum‐deposited perovskite EMLs, we fabricated vacuum‐deposited PeLEDs (**Figure** **4**a) from these EMLs with the device configuration indium tin oxide (ITO, 110 nm)/1,4,5,8,9,11‐hexaazatriphenylene hexacarbonitrile (HAT‐CN, 10 nm)/TAPC (40 nm)/CzSi (0 or 5 nm)/ perovskite (50 nm)/2,2′,2′′‐(1,3,5‐benzinetriyl)tris(1‐phenyl‐1*H*‐benzimidazole) (TPBi, 50 nm)/lithium fluoride (LiF, 1 nm)/Al (120 nm). Because the highest occupied molecular orbital (HOMO) of CzSi was lower in energy relative to those of TAPC and perovskite, we limited the thickness of the CzSi film to 5 nm to minimize its effect on impeding hole transport. Figure 4b presents the current–voltage–luminescence (*J*–*V*–*L*) characteristics of the PeLEDs prepared with and without a CzSi under‐layer. The turn‐on voltage of the PeLED with the CzSi under‐layer was slightly higher than that of the device without the CzSi under‐layer. Nevertheless, the device with the under‐layer exhibited a superior EQE (2.54%) and value of *L*_max_ (16200 cd m^−2^, operated at 11 V) when compared with those of the underlayer‐free device (EQE = 0.74%; *L*_max_ = 11500 cd m^−2^), suggesting fluent hole transport through the ultrathin (5 nm) CzSi layer (Figure 4c). The two types of PeLED provided almost the same electroluminescence (EL) spectra (Figure S6a, Supporting Information), indicating that the composition of the perovskite layer was not changed by the presence of the under‐layer, consistent with the PL data. We further improved the device performance by optimizing the layer thickness and employing an electron transport layer (ETL) with a lower‐energy lowest unoccupied molecular orbital (LUMO), to balance the charge transport, with the configuration ITO (110 nm)/HAT‐CN (5 nm)/TAPC (20 nm)/CzSi (5 nm)/ perovskite (30 nm)/2,4,6‐tris(2‐(1*H*‐pyrazol‐1‐yl)phenyl)‐1,3,5‐triazine (3P‐T2T, 30 nm)/LiF (1 nm)/Al (120 nm). The device exhibited a low turn‐on voltage of 2.1 V, an EQE of 4.1%, and a value of *L*_max_ of 11000 cd m^−2^ at 5.3 V (Figure 4d,e, blue). Finally, we integrated an ultrathin GABr upper‐layer into this vacuum‐deposited PeLEDs. In our earlier study of the PL intensities of EMLs prepared with GABr, we found that the highest PL efficiency occurred when using GABr at concentrations greater than or equal to 2.0 mg mL^−1^. Nevertheless, the PeLEDs incorporating EMLs modified with high concentrations of GABr provided irregular *J*–*V* characteristics and displayed very poor device performance, without any detectable emission (Figure S6b, Supporting Information). High surface roughness of the perovskite thin film treated with a high GABr concentration was observed (Figure S7, Supporting Information). The rough surface could not be evenly covered by the later deposited upper‐layers, and thus, damaged the device performance. The EML modified with a moderate amount of GABr (0.75 mg mL^−1^); however, provided a vacuum‐sublimed PeLED demonstrating distinguished performance, with EQEs as high as 10.9% and a CE of 38.7 cd A^−1^ (Figure 4d,e, red). This vacuum‐deposited inorganic PeLEDs is the first to have achieved EQEs exceeding the 10% milestone.

Notably, the luminance of the PeLED with a GABr upper‐layer decreased when the driving voltage was greater than 3 V (Figure 4d, red). This behavior was improved after introducing an ultrathin (0.5 nm) layer of 2,8‐bis(diphenylphosphoryl)dibenzo[*b*,*d*]thiophene (PPT), after depositing the GABr layer. According to the analysis of trap‐state density from the current density–voltage characteristics employing space‐charge limited current (SCLC) method (Figure S8, Supporting Information), the ultrathin PPT layer effectively suppressed the surface defects of the perovskite with GABr upper‐layer, and thus eliminated the luminance quenching. The deep HOMO energy level (6.7 eV) of PPT was also useful for hole blocking. The energy level diagram of the PeLEDs is shown in Figure S9a in the Supporting Information. The valance level of the perovskite was determined by ultraviolet photoelectron spectroscopy (UPS) spectra (Figure S9b, Supporting Information) and the energy bandgap was extracted from the absorption spectrum (Figure S9c, Supporting Information). The optimized PeLED having the structure ITO (110 nm)/HAT‐CN (5 nm)/TAPC (20 nm)/CzSi (5 nm)/perovskite (30 nm)/GABr (5 nm)/PPT (0.5 nm)/3P‐T2T (30 nm)/LiF (1 nm)/Al (120 nm) provided an EQE of 10.4% and a CE of 38.2 cd A^−1^ (Figure 4e, orange). Although the EQE of this device was slightly lower than that of the corresponding device prepared without PPT, the phenomenon of luminance quenching had been eliminated to some degree. The device exhibited a value of *L*_max_ (11800 cd m^−2^ at 5.4 V; Figure 4d, orange) that was three times higher than that of the device prepared without PPT (3700 cd m^−2^). At this stage, we fine‐tuned the molar ratio of CsBr and PbBr_2_ once again, after having introduced the ultrathin CzSi under‐layer and GABr and PTT upper‐layers. The value of *L*_max_ of the PeLED reached 75 300 cd m^−2^ at 6 V after slightly decreasing the CsBr/PbBr_2_ molar ratio to 1.37, leading better overall charge balance and maintaining an EQE of 8.55% at 3.7 V (Figure S6c,d, Supporting Information). The performance of this vacuum‐deposited PeLED remains quite competitive, but its value of *L*_max_ is four times higher than that of the best vacuum‐deposited PeLEDs reported previously.^[^ ^58^ ^]^

Notably, the vacuum‐deposited perovskite thin films and devices featured excellent, smooth, and homogeneous surface morphologies, ideal for large area manufacturing and device reproducibility. Unfortunately, a smooth morphology implies a moderate light‐outcoupling efficiency, due to a lack of rough structures for light scattering. Hence, we integrated a hemispherical lens onto the PeLED to function as a light‐outcoupling enhancer. The device presenting the hemispherical lens provided extraordinary performance characteristics, with a value of EQE_max_ of 21.1% (Figure 4f,g)—again, the highest reported performance for a vacuum‐deposited PeLED with a light outcoupling booster. The device performance enhancement (approximately twofold) by the light‐outcoupling enhancer corresponded well with the theoretical simulation as shown in Figure S10. To showcase the high reproducibility of these vacuum‐deposited PeLEDs, we measured the performance data from 24 devices in two batches; Figure S11 (Supporting Information) plots their statistical EQEs. The revealed narrow spread in device performance would be very difficult, if not impossible, to achieve in solution‐processed counterparts. We measured the preliminary device lifetimes of the PeLEDs prepared with and without GABr upper‐layers at an initial luminance of 100 cd m^−2^ under N_2_. The GABr upper‐layer device displayed a rapid increase in luminance, which remained roughly constant for 40 min. The device eventually exhibited a T50 lifetime of 3.2 h (Figure 4h)—a fourfold improvement over that of the device prepared without a GABr upper‐layer. These results explicitly demonstrate the benefits of a GABr upper‐layer in improving the performance and stability of PeLEDs. Nevertheless, over‐shooting of device efficiency was still observed in the PeLEDs operational lifetime measurement, indicating that there was still ion migration in the devices, and left a room for improvement.^[^ ^59^, ^60^ ^]^

## 3 Conclusion

Although vacuum‐deposited amorphous EMLs in OLEDs are typically insensitive to their under‐ and upper‐layers, in this study we observed the opposite behavior for vacuum‐sublimed perovskite EMLs, with the emission enhanced tenfold after insertion of an ultrathin (5 nm) CzSi under‐layer. The presence of this CzSi layer inhibited Pb^0^ from forming inside the EMLs; in contrast, Pb^0^ was deposited on conventional TAPC HTLs. The device efficiency improved further, by 2.5‐fold, after introducing a GABr upper‐layer. The quasi‐2D structure prepared with the GABr upper‐layer appeared to passivate the vacuum‐deposited EMLs, and minimized the number of unwanted nonradiative recombination pathways. The maximum achievable brightness of the PeLEDs then increased again after incorporating a second ultrathin (0.5 nm) upper‐layer of PPT to suppress surface defects of the perovskite with the GABr upper‐layer. These hierarchical strategies realized vacuum‐deposited PeLEDs with high EL efficiency, with EQEs as high as 10.9 and 21.1% achieved in the absence and presence, respectively, of a light out‐coupling structure, breaking through the 10% EQE milestone. Furthermore, we obtained a value of *L*_max_ of 75 300 cd m^−2^ and promising device reproducibility. We believe that the study paves the way toward realizing efficient vacuum‐evaporated PeLEDs with performance characteristics comparable with those of solution‐process devices.

## 4 Experimental Section

### Materials

CsBr (99.999%) and 2‐propanol (IPA, anhydrous, 99.5%) were purchased from Sigma–Aldrich. PbBr_2_ (>98%) was purchased from TCI. GABr (>98%) was purchased from Greatcell Solar. HAT‐CN, TAPC, CzSi, PPT, and 3P‐T2T were purchased from Lumtec.

### Device Preparation

All organic materials were purified through thermal gradient sublimation prior to use. Precleaned ITO‐coated glasses were used as substrates. The substrates were loaded into a vacuum chamber at a base pressure of 3×10^–6^ torr and kept at room temperature when depositing the organic and perovskite layers sequentially. In the optimized vacuum‐deposited PeLED structure, HAT‐CN, TAPC, and CzSi served as the hole injection layer, hole transport layer, and under‐layer, respectively; they were deposited sequentially on the substrates. To obtain a perovskite layer with a CsBr/PbBr_2_ molar ratio of 1.4:1, CsBr was deposited at a rate of 1.22 Å s^−1^, while PbBr_2_ was deposited at a rate of 1 Å s^−1^. During codeposition, the deposition rates were monitored using three quartz crystal microbalance sensors; the first sensor monitored the deposition of the lead halide, the second monitored the deposition of the cesium halides, and the third monitored the total deposition. After codeposition of the perovskite layer, the samples were transferred into a N_2_‐filled glove box without exposure to air and then a GABr (0.75 mg mL^−1^ in IPA) upper‐layer was deposited through dynamic spin‐coating (6000 rpm, 60 s). The samples were returned to the high‐vacuum chamber for the deposition of PPT, 3P‐T2T, LiF, and Al. Here, PPT and 3P‐T2T served as the interlayer for electron injection and the electron transport layer, respectively, while LiF and Al served as the cathode of the PeLED. The final optimized device had the configuration glass substrate/ITO (110 nm)/HAT‐CN (5 nm)/TAPC (20 nm)/CzSi (5 nm)/perovskite (30 nm)/GABr (5 nm)/PPT (0.5 nm)/3P‐T2T (30 nm)/LiF (1 nm)/Al (120 nm). The device area (0.01 cm^−1^) was defined by the overlap of the ITO anode and the Al cathode. All film thicknesses were confirmed through ellipsometry (V‐VASE, J. A. Woollam).

### Characterization

UV–vis absorption spectra were acquired using a UV–vis spectrophotometer (UV‐2600, Shimadzu) with an integrating sphere. PL spectra were recorded using a spectrometer (Flame, Ocean Optics) with a 365‐nm LED as the pumping source. TRPL spectra were measured using the time‐correlated single photon counting method, with a 375‐nm diode laser (LDH‐P‐C‐375 M, PicoQuant) as the pumping source. The laser fluence used in the TRPL measurements was around 2.87nJcm^−2^ per pulse. After the consideration of thin film absorbance (O.D. = 0.18), thickness (50 nm) and the photon energy of 375 nm, the photoinduced carrier density is ≈3.68 × 10^14^cm^−3^ per pulse, which can be considered as a low laser fluence for PL lifetime to evaluate the nonradiative recombination process. XRD patterns were recorded using a Bruker D2 PHASER with Cu K*α* radiation. SEM images were recorded using a Hitachi‐SU8010 scanning electron microscope. XPS and UPS spectra were recorded with electron spectroscopy for chemical analysis (PHI 5000 Versaprobe II, ULVAC‐PHI). Surface morphologies were characterized using AFM (Dimension ICON, Bruker). TEM images were recorded using a spherical‐aberration‐corrected field‐emission transmission electron microscope (Cs‐corrected TEM, JEOL ARM‐200FTH) at an accelerating voltage of 200 kV. Elemental distributions were measured using equipped EDS. TA measurements were conducted by using a home‐made system that can flexibly support excitation at 429 nm and probe spectrum across 420–550 nm.^[^ ^61^, ^62^, ^63^ ^]^ The *J–V–L* characteristics of devices in a glove box were measured simultaneously using a 2636A source meter (Keithley Instruments) as a driving source, while the total forward luminous flux was measured by an integrating sphere system with a calibrated Si photodetector.^[^ ^64^ ^]^ Luminance was calculated assuming a Lambertian emission characteristic of the devices. The EL spectra were recorded using a spectrometer (Flame, Ocean Optics). The optical simulation of PeLEDs was done with SETFOS (FLUXiM). For measurement of the absorption, PL intensity, TRPL, XPS, UPS, SEM, and AFM characteristics, perovskite thin films were fabricated under the same conditions as those used to obtain the emission layers of the PeLEDs. For XRD measurements, the perovskite films were deposited on Si substrates at a greater thickness (50 nm) to obtain more obvious signals.

## Conflict of Interest

The authors declare no conflict of interest.

## Supporting information

Supporting Information

## Data Availability

The data that support the findings of this study are available from the corresponding author upon reasonable request.
