# Supplementary material for: Vacuum‐Deposited Inorganic Perovskite Light‐Emitting Diodes with External Quantum Efficiency Exceeding 10% via Composition and Crystallinity Manipulation of Emission Layer under High Vacuum
Source: Adv Sci (Weinh). 2023 Feb 7;10(10):2206076. doi: 10.1002/advs.202206076 (PMC10074115; doi:10.1002/advs.202206076)
Supplement: Supplementary file 1 — Supporting Information [file ADVS-10-2206076-s001.pdf]

Supporting Information

Vacuum-Deposited Inorganic Perovskite Light-Emitting Diodes with External Quantum Efficiency Exceeding 10% via Composition and Crystallinity Manipulation of Emission Layer under High Vacuum

Chung-An Hsieh, Guang-Hsun Tan, Yung-Tang Chuang, Hao-Cheng Lin, Po-Ting Lai, Pei-En Jan, Bo-Han Chen, Chih-Hsuan Lu, Shang-Da Yang, Kai-Yuan Hsiao, Ming-Yen Lu, Li-Yin Chen and Hao-Wu Lin**

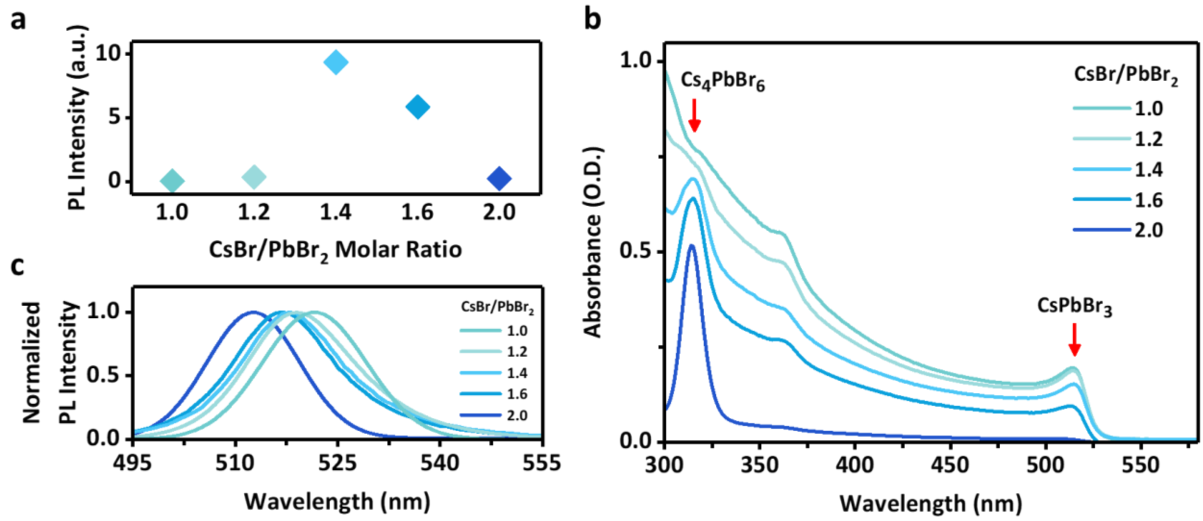

Figure S1. **a,** PL intensity, **b,** absorption spectra, and **c,** normalized PL intensity peaks of perovskite thin films fabricated with various $CsBr/PbBr_2$ molar ratios.

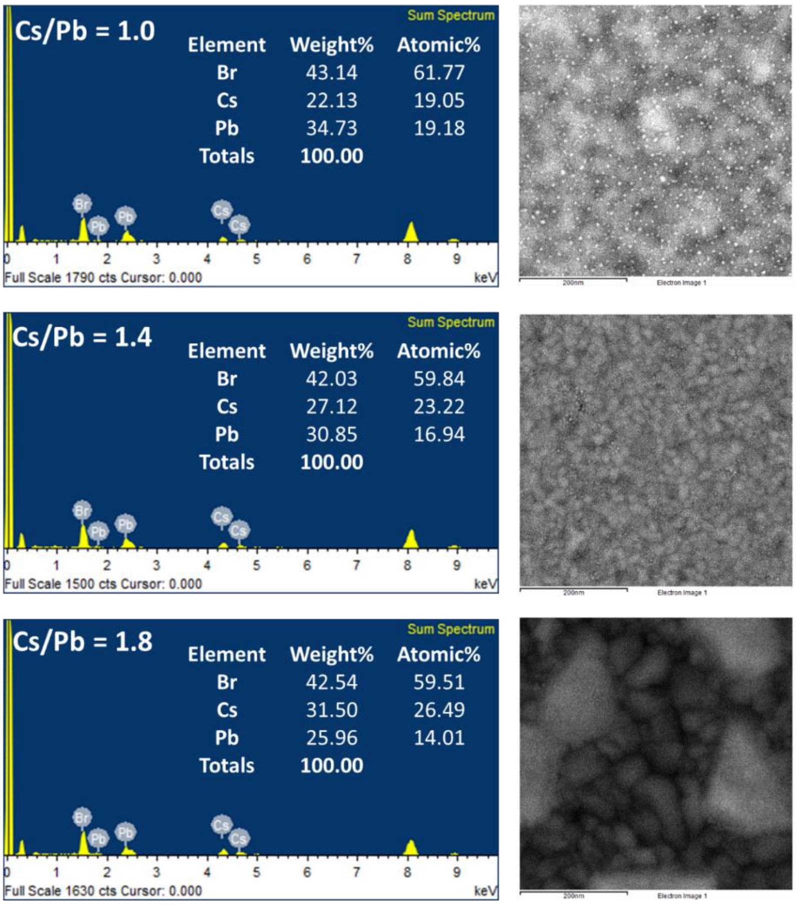

Figure S2. EDS results obtained from perovskite thin films fabricated with various $CsBr/PbBr_2$ molar ratios.

Table S1. The comparison between the molar ratios of experiment conditions and EDS results.

	**Molar ratio ($CsBr/PbBr_2$)**		
**Exp. condition**	1.0	1.4	1.8
**EDS data**	0.99	1.37	1.89

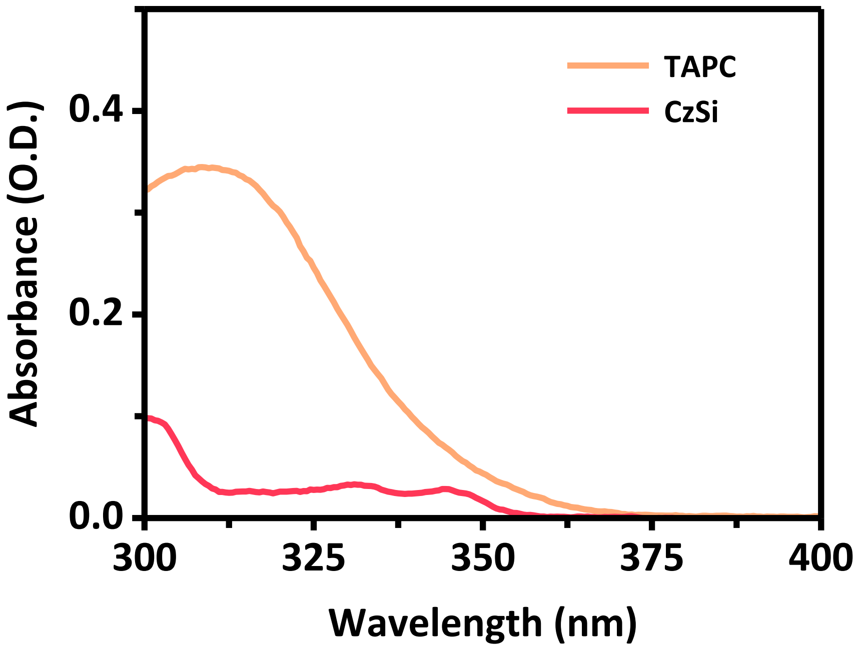

Figure S3. Absorption spectra of 50-nm-thick pristine TAPC and CzSi films.

Table S2. TRPL data for perovskite thin films prepared with different under-layer materials, extracted by fitting with a triexponential decay formula.

Under-layer Material	τ_1 [ns] [a)]	A_1 (%)	τ_2 [ns]	A_2 (%)	τ_3 [ns]	A_3 (%)	Average lifetime, τ_a [ns] [b)]
**TAPC**	0.7	97.0	5.0	3.0	–	–	1.5
**UGH-2**	0.6	88.0	5.1	10.3	47.6	1.7	22.3
**CzSi**	0.6	83.9	4.8	14.0	49.0	2.1	24.4

a) PL lifetimes were fitted using the exponential decay equation below:

$$y = y_0 + A_1 \exp\left(-\frac{x}{\tau_1}\right) + A_2 \exp\left(-\frac{x}{\tau_2}\right) + A_3 \exp\left(-\frac{x}{\tau_3}\right)$$

b) Average lifetimes were calculated using the equation below:

$$\tau_a = \frac{\sum A_i \tau_i^2}{\sum A_i \tau_i}$$

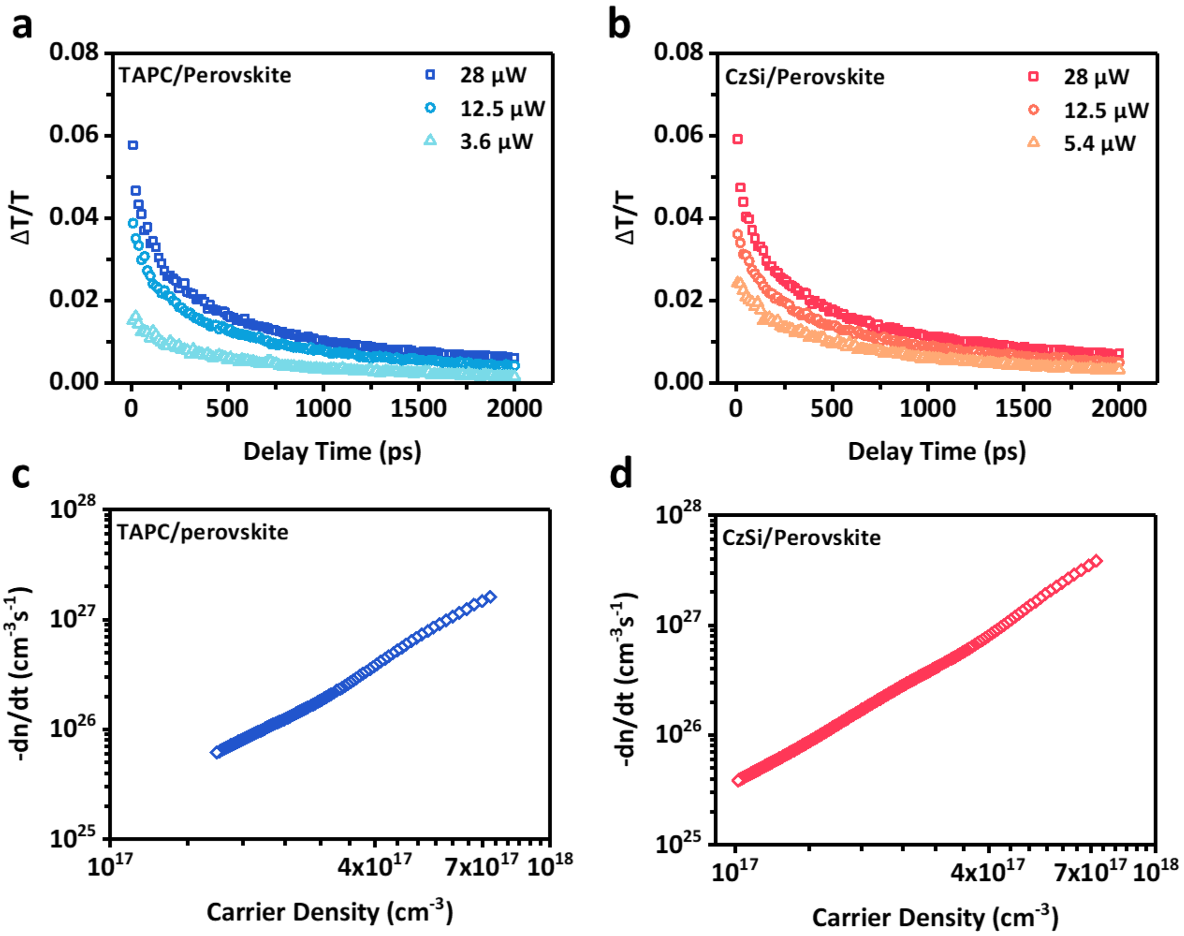

Figure S4. **a,b,** TA kinetics under various excitation fluences. **c,d,** The dependence of carrier recombination decay rates on carrier density of perovskite thin films prepared with different under-layers.

Table S3. The fitted results of recombination rate constants of perovskite thin films prepared with different under-layers.

Under-layer Material	*a* (s^{-1})	*b* ($cm^3\ s^{-1}$)	*c* ($cm^6\ s^{-1}$)
**TAPC**	6.67×10^{8}	1.54×10^{-10}	3.65×10^{-27}
**CzSi**	4.08×10^{7}	1.69×10^{-9}	8.19×10^{-27}

Table S4. TRPL data for perovskite films treated with different concentrations of GABr, extracted by fitting with a triexponential decay formula.

**GABr concentration (mg/mL)**	τ_1 [ns]	A_1 (%)	τ_2 [ns]	A_2 (%)	τ_3 [ns]	A_3 (%)	**Average lifetime, τ_a [ns]**
**0**	0.8	96.7	10.5	3.0	109.7	0.3	24.0
**0.5**	1.2	94.2	12.9	4.8	104.9	1.0	36.9
**0.75**	1.5	91.5	13.3	7.5	106.4	1.0	40.3
**1.0**	1.7	79.6	10.9	17.5	84.1	2.9	41.1
**2.0**	1.6	68.7	12.2	25.3	84.9	6.0	51.9

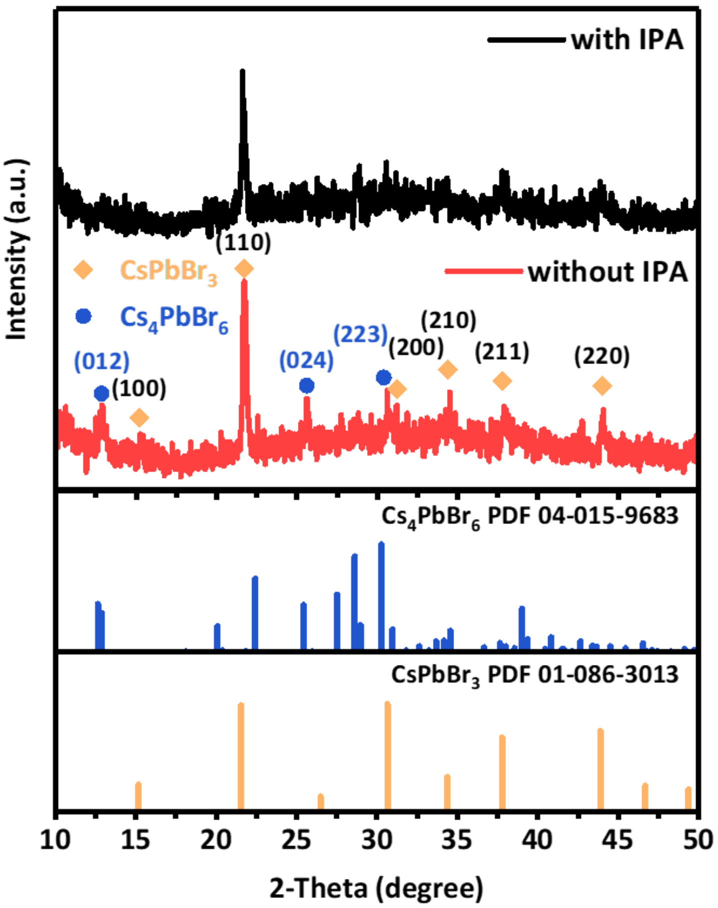

Figure S5. XRD patterns of perovskite films prepared with and without IPA treatment.

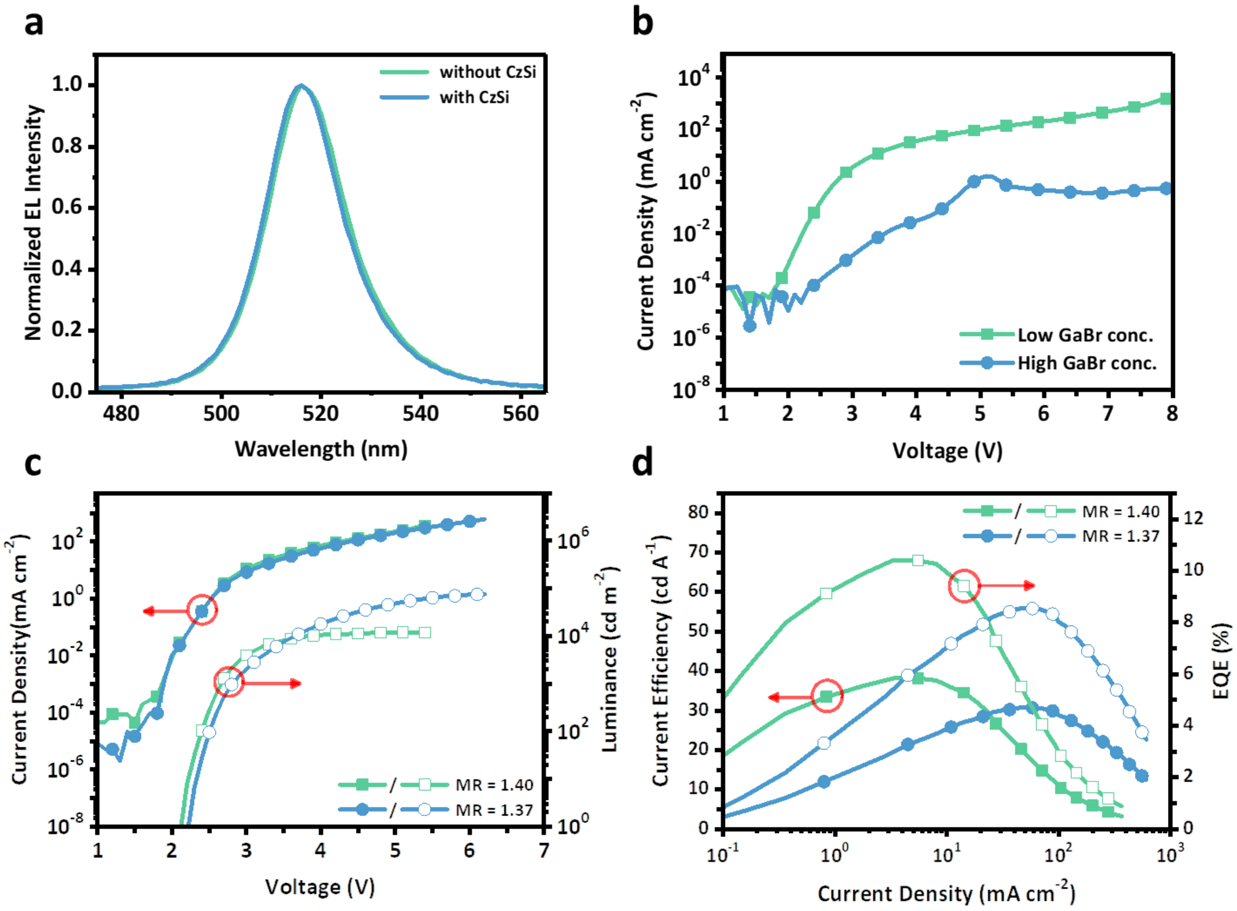

Figure S6. **a,** EL spectra of PeLEDs prepared with and without CzSi. **b,** *J*–V curves of PeLEDs treated with high (≥2.0 mg/mL) and low (0.75 mg/mL) concentrations of GABr; the former displayed irregular *J*–V characteristics and very poor device performance, compared with the latter. **c,** *J*–V–L characteristics and **d,** CE and EQE data of PeLEDs prepared with various $CsBr_2/PbBr_2$ molar ratios in the perovskite EML.

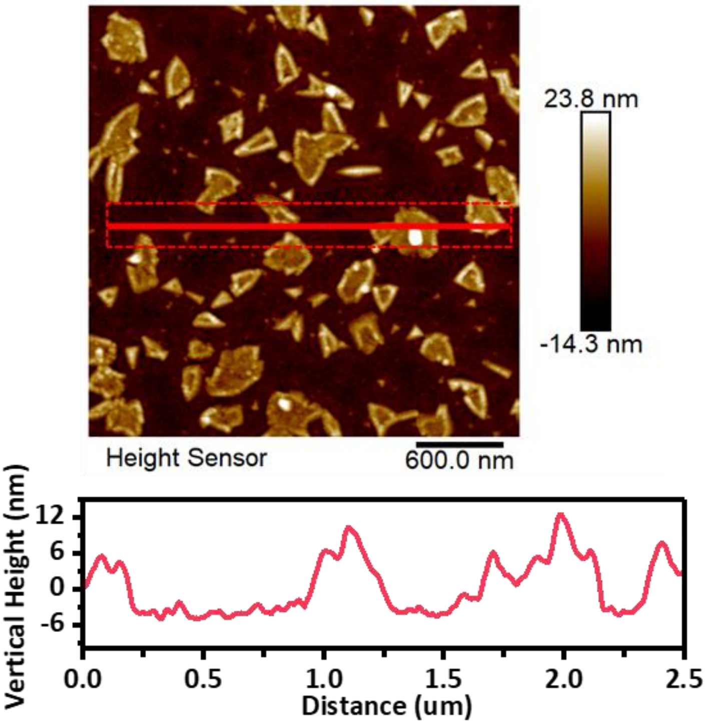

Figure S7. AFM image and vertical height profile of perovskite film prepared with high (2.0 mg/mL) concentration of GABr.

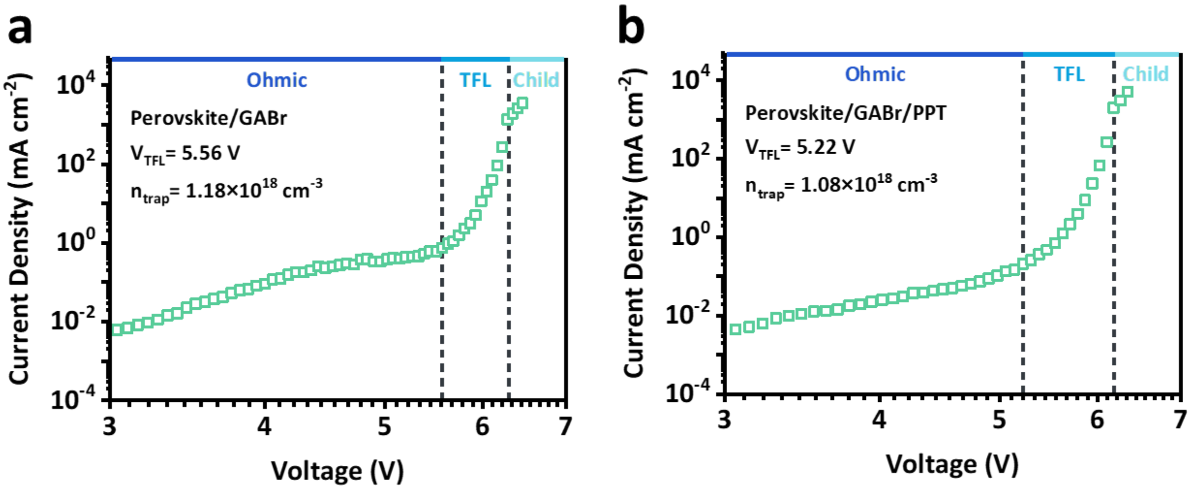

Figure S8. a, b, *J*–V characteristics of electron-only devices without and with PPT upper-layer.

The electron-only devices configuration of ITO/LiF/3P-T2T/perovskite/GABr/with or without PPT/3PT-2T/LiF/Al. The trap-state density (n_{trap}) can be determined by the trap-filled-limit voltage (V_{TFL}) based on the equation below:

$$V_{TFL} = n_{trap} \frac{ed^2}{2\varepsilon\varepsilon_0}$$

where the d is the thickness of perovskite (50 nm) and the value of ε for $CsPbBr_3$ is 4.8.[S1]

References

[S1] Y. Dong, Y.-K. Wang, F. Yuan, A. Johnston, Y. Liu, D. Ma, M.-J. Choi, B. Chen, M. Chekini, S.-W. Baek, L. K. Sagar, J. Fan, Y. Hou, M. Wu, S. Lee, B. Sun, S. Hoogland, R. Quintero-Bermudez, H. Ebe, P. Todorovic, F. Dinic, P. Li, H. T. Kung, M. I. Saidaminov, E. Kumacheva, E. Spiecker, L.-S. Liao, O. Voznyy, Z.-H. Lu, E. H. Sargent, *Nat. Nanotechnol.* 2020, 15, 668.

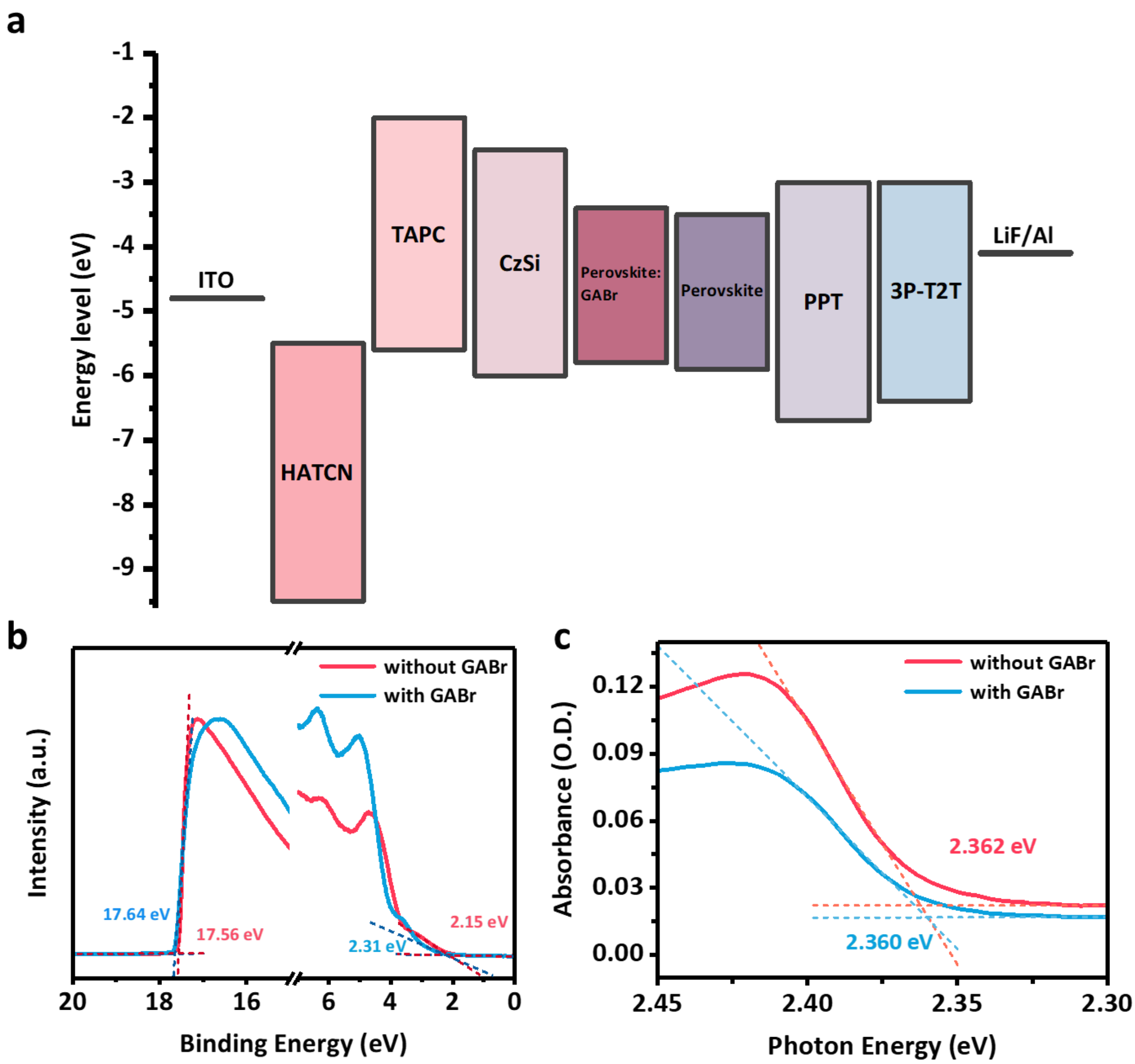

Figure S9. **a,** The energy level diagram of the PeLEDs. **b,** UPS spectra and **c,** absorption spectra of perovskite films prepared with and without GABr treatment.

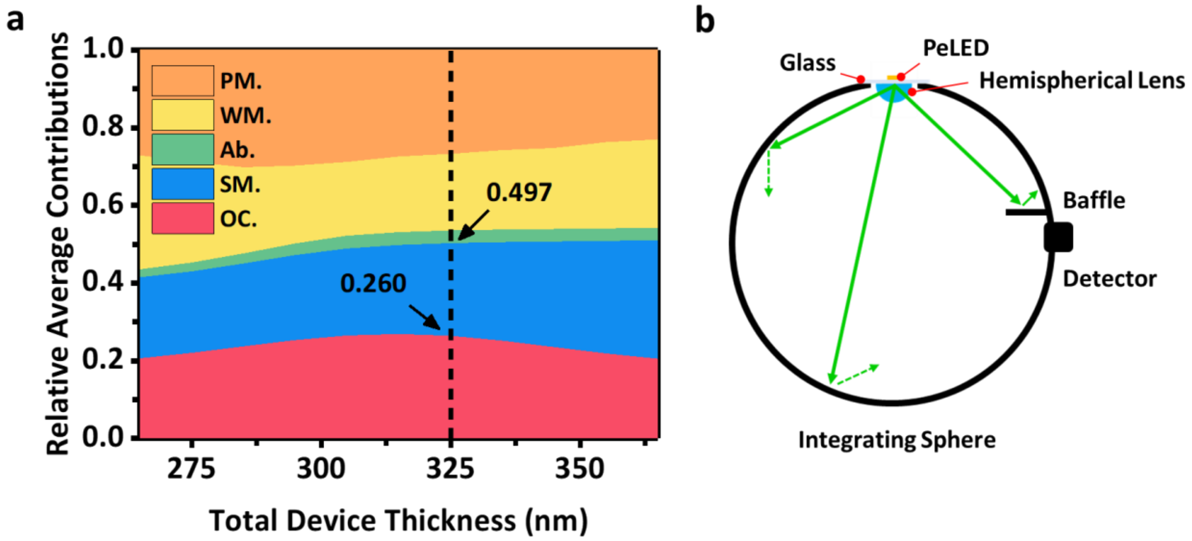

Figure S10. **a,** Optical simulation of PeLED with various total device thickness. The out-coupling ratio of the planar device is ~ 26.0% while the out-coupling ratio of the device with a hemi-sphere is ~ 49.7%. **b,** Schematic diagram of the integrating sphere measurement setup.

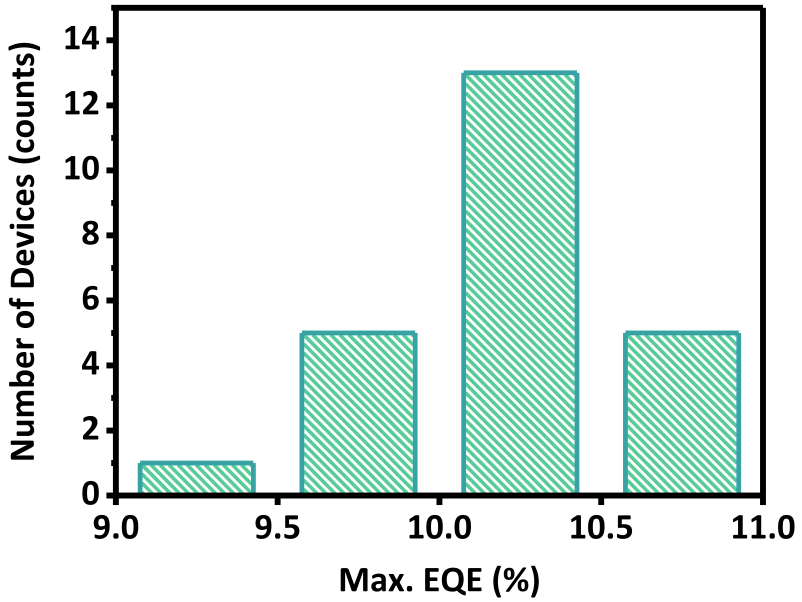

Figure S11. Histogram of EQE values from 24 devices prepared in two batches.
